# Analysis of Wide-Lane Ambiguities Derived from Geometry-Free and Geometry-Based Precise Point Positioning Models and Their Implication for Orbit and Clock Quality

**DOI:** 10.3390/s18061760

**Published:** 2018-05-31

**Authors:** Gang Chen, Sijing Liu, Qile Zhao

**Affiliations:** 1 College of Maritime Science and Technology, China University of Geosciences, Wuhan 430074, China; ddwhcg@cug.edu.cn; 2 Institute of Geophysics and Geomatics, China University of Geosciences, Wuhan 430074, China; 3 GNSS Research Center, Wuhan University, Wuhan 430079, China

**Keywords:** geometry-free, geometry-based, wide-lane ambiguity, orbit and clock residual error

## Abstract

Orbit and clock products are used in real-time global navigation satellite systems (GNSS) precise point positioning (PPP) without knowing their quality. This study develops a new approach to detect orbit and clock errors through comparing geometry-free and geometry-based wide-lane ambiguities in a PPP model. The reparameterization and estimation procedures of the geometry-free and geometry-based ambiguities are described in detail. The effects of orbit and clock errors on ambiguities are given in analytical expressions. The numerical similarity and differences of geometry-free and geometry-based wide-lane ambiguities are analyzed using different orbit and clock products. Furthermore, two types of typical errors in orbit and clock are simulated and their effects on wide-lane ambiguities are numerically produced and analyzed. The contribution discloses that the geometry-free and geometry-based wide-lane ambiguities are equivalent in terms of their formal errors. Although they are very close in terms of their estimates when the used orbit and clock for geometry-based ambiguities are precise enough, they are not the same, in particular, in the case that the used orbit and clock, as a combination, contain significant errors. It is discovered that the discrepancies of geometry-free and geometry-based wide-lane ambiguities coincide with the actual time-variant errors in the used orbit and clock at the line-of-sight direction. This provides a quality index for real-time users to detect the errors in real-time orbit and clock products, which potentially improves the accuracy of positioning.

## 1. Introduction

Precise point positioning (PPP) using global navigation satellite systems (GNSS) measurements was initially proposed in [1] in order to solve the high computation burden of the network approach. The PPP technique only employs a single receiver to determine its position without explicitly using reference stations. To do so, precise orbit and clock are needed to enable absolute positioning of high accuracy [2]. This technique has been intensively developed from static to kinematic positioning [3], from post-processing to real-time service [4,5], from using global positioning system (GPS) only to using multi-constellation systems [6], from ambiguity-float to integer ambiguity-fixed solution [7,8,9,10], from decimeter to centimeter accuracy [11]. The PPP technique started with the ionosphere-free combination approach [1] and then developed with the raw observation approach [11,12,13,14,15]. One of the major differences of these two approaches is that one eliminates the ionosphere delay while the other estimates it. Another main difference is that how to deal with the ambiguity parameter. The ambiguity term estimated with the ionosphere-free approach is actually a combination of narrow-lane (NL) and wide-lane (WL) ambiguity, while the raw observation approach produces L1 and L2 ambiguity parameters simultaneously together with the ionosphere term (let us consider only the case of dual frequencies). The common point for these two approaches is that their ambiguity parameters do not have integer nature, as a double-difference cannot be formed using a single receiver. In this case, the satellite hardware delay cannot be separated or eliminated from the ambiguities in the PPP mode. The term takes typically several tens of minutes to converge when the positioning starts or resets after data interruption. The accuracy can be improved, and the convergence time can be shortened if the ambiguities can be fixed correctly to their integers using additional corrections, such as satellite phase biases. 

A key task for the PPP integer ambiguity resolution is to separate or identify satellite phase biases in the ambiguity term in the reference network and then to apply these biases to their corresponding ambiguities in mobiles to restore integer nature [13,14]. The ionosphere-free approach basically uses two parallel processes [7,8,9,10]. One process is to compute the geometry-free (GF) Hatch-Melbourne-Wübbena combinations [16,17,18] in order to produce WL satellite phase biases and integers. The other process is to carry out the geometry-based (GB) positioning to compute the ionosphere-free ambiguity terms and further separate NL biases with involvement of integers of GF WL ambiguities. The raw observation approach uses only one process that produces L1 and L2 ambiguities simultaneously [14], which are then used to form GB WL ambiguities and further produce WL satellite phase biases. A variant of the raw observation approach is to produce WL and L2 ambiguities simultaneously in the GB model [15]. WL ambiguities are the basis of going forwards for these two approaches. The difference is that one produces them with the GF model while the other with the GB model. Then the question raises: are the GF WL ambiguities the same or equivalent with the GB ones from even a single receiver in terms of estimates themselves and their formal precision? The reason for such a question is to clear off the hesitation of mixing use of the corrections. That is to say, if they are not the same or equivalent, one may hesitate to apply the GB WL corrections to the ionosphere-free approach, or apply the GF WL corrections to the raw observation approach in user side. The question has been positively answered in an analytical way in [19] using a between-satellite single-difference functional model of multiple stations. However, there is no numerical analysis based on the real data in literature. It concludes that the GF WL ambiguity precision does not differ too much from its GB counterpart under an assumption of small to regional networks [19]. This assumption does not consider the orbit and clock residual errors (i.e., the remaining error in precise orbits and clocks as they are not perfect). Different questions may then raise. Do the orbit and clock residual errors make differences for the GF and GB ambiguities? This is certainly not the case for the GF ambiguities as the geometry related parameters, such as satellite/receiver positions, satellite/receiver clock as well as troposphere delay, are eliminated. Then how about the GB ambiguities? If so, in what level of orbit and clock quality, one could claim that the GF and GB ambiguities are equivalent? 

The quality of orbit and clock has been improved in the past years for post-processing products [20] and real-time products [21]. The majority of the GNSS community is producing real-time orbit and clock data step by step [5,6]. A set of satellite orbit parameters (initial state and solar radiation pressure) is first determined using a network of globally distributed reference stations using the past 24–48 h of GNSS measurements. The orbits of at least 3 h are predicted using the obtained orbit parameters. The predicted orbits are then used as input for clock bias filtering in which real-time observations are used. The accuracy of the predicted orbit is dominated by the solar radiation pressure [6]. In addition, the predicted orbit may not be precise enough in the eclipse and just out of the eclipse seasons, due to the fact that the yaw attitude is not properly modelled. It is obvious that the remaining error of predicted orbits with longer predict arc length could make significant negative impact on the GB NL ambiguity estimation and integer resolutions [6]. Their impact on GB WL ambiguities remains unanalyzed. Although it is possible to reduce the orbit error by shortening the prediction length, the variation of errors could range from a couple of centimeters to decimeters. There are also exceptional occasions where some satellites, like GPS IIF, could have large errors up to meters in their predicted orbits [22]. These errors could significantly worsen the clock bias estimation and satellite phase bias estimation as well as PPP integer ambiguity resolution in user side, in particular for real-time users.

It is common to evaluate the quality of real-time orbit and clock by comparing them with the post-processing counterpart separately [21]. However, the post-processing products are available only after one day or a couple of hours later, which is not very acceptable for real-time users. In addition, it is well known that the clock products absorb partly the errors of predicted orbits, particularly errors of the line-of-sight (LOS) direction [23,24,25]. Therefore, it is more reasonable to assess orbit and clock residual error as a whole directly from a user’s perspective. The differences between GF and GB wide-lane ambiguities is a starting point to investigate. 

This contribution aims at analysis of single-site ambiguities derived from both GF and GB model. We focus on numerical computation and analysis using raw observations without differential and combination operator in the measurement domain. The GB WL ambiguities are derived using different orbit and clock products. To simplify the presentation and discussion, we are focusing on GPS in this contribution, but this does not affect the generality of our analysis for other constellations. We also investigate the discrepancies between GF and GB WL ambiguities to disclose the orbit and clock quality from a user’s perspective. The contribution is arranged as follows: First, the GF and GB models are respectively presented in detail. Second, the effects of orbit and clock errors on the ambiguities are given in an analytical way. Third, the results of GB and GF ambiguities are numerically analyzed for both post-processing and real-time orbit and clock products. In addition, we simulate two types of errors in orbit and clock and reprocess the data and investigate the impact on WL ambiguities for further understanding. Finally, the study is summarized with some conclusions.

## 2. WL Ambiguities Derived from the GF Model

The observation equation for measurements of code *p* and phase φ tracked by a receiver at location *r* from GPS satellites s (s=1, 2, ⋯, m), at frequency i (i=1, 2) at epoch *k*, can be expressed in meters as follows [26]:

(1) $$\begin{array}{l} E\{p_{i,r}^{s}(k)\}=\rho_{r}^{s}(k)+m_{r}^{s}\cdot T_{r}+\delta \;t_{r}(k)-\delta \;t^{s}(k)+\mu_{i}I_{r}^{s}(k)+d_{p_{i},r}-d_{p_{i}}^{s}\\ E\{\varphi_{i,r}^{s}(k)\}=\rho_{r}^{s}(k)+m_{r}^{s}\cdot T_{r}+\delta \;t_{r}(k)-\delta \;t^{s}(k)-\mu_{i}I_{r}^{s}(k)+d_{\varphi_{i},r}-d_{\varphi_{i}}^{s}+\lambda_{i}N_{i,r}^{s} \end{array}$$

where E{⋅} denotes the expectation operator; $\rho_{r}^{s}(k)$ is the geometric range between the receiver and satellite; $T_{r}$ the zenith tropospheric delay with mapping function $m_{r}^{s}$; $\delta \;t_{r}(k)$ and $\delta \;t^{s}(k)$ the receiver and satellite clock bias in meters, respectively; $\mu_{i}=\lambda_{i}^{2}/\lambda_{1}^{2}$, $\lambda_{i}$ the carrier wavelength of frequency *i*; $I_{r}^{s}(k)$ the ionospheric delay; $d_{p_{i},r}^{}$ and $d_{\varphi_{i},r}$ are the receiver hardware delays (or biases) on code and phase, respectively; $d_{p_{i}}^{s}$ and $d_{\varphi_{i}}^{s}$ are the satellite hardware delays (or biases) on code and phase, respectively; where it is assumed that these satellite hardware delays are relatively stable in time; $N_{i,r}^{s}$ the integer carrier ambiguity. All terms with (k) are supposed to be significantly changed in time.

The so-called GF observation model dispenses with geometric a priori information. The geometry part is obtained by combining all non-dispersive terms, including the geometric range $\rho_{r}^{s}(k)$, satellite $\delta \;t^{s}(k)$ and receiver clock $\delta \;t_{r}(k)$ and tropospheric delay $m_{r}^{s}\cdot T_{r}$, even though the system is rank deficient. Not all terms are estimable. The hardware delays here are chosen as S-basis, absorbed by other terms [19]. Let us define the geometry part as one common term for all observations from one satellite:

(2) $$R_{r}^{s}(k):=\rho_{r}^{s}(k)+m_{r}^{s}\cdot T_{r}+\delta \;t_{r}(k)-\delta \;t^{s}(k)+\frac{\mu_{2}}{\mu_{2}-\mu_{1}}(d_{p_{1},r}-d_{p_{1}}^{s})-\frac{\mu_{1}}{\mu_{2}-\mu_{1}}(d_{p_{2},r}-d_{p_{2}}^{s})$$

and the ionosphere term is also combined with part of satellite and receiver hardware biases:

(3) $${\tilde{I}}_{r}^{s}(k):=I_{r}^{s}(k)+\frac{\mu_{1}}{\mu_{2}-\mu_{1}}(d_{p_{2},r}-d_{p_{2}}^{s}-d_{p_{1},r}+d_{p_{1}}^{s})$$

and the *ambiguity* terms for L1 and L2 frequency are defined as:

(4) $${\tilde{N}}_{i,r}^{s}:=N_{i,r}^{s}+\frac{1}{\lambda_{i}}\left\{d_{\varphi_{i},r}-d_{\varphi_{i}}^{s}+\frac{\mu_{i}+\mu_{1}}{\mu_{2}-\mu_{1}}(d_{p_{2},r}-d_{p_{2}}^{s})-\frac{\mu_{i}+\mu_{2}}{\mu_{2}-\mu_{1}}(d_{p_{1},r}-d_{p_{1}}^{s})\right\}$$

Therefore, the *functional GF model* is written as:

(5) $$\begin{array}{l} E\{p_{i,r}^{s}(k)\}=R_{r}^{s}(k)+\mu_{i}{\tilde{I}}_{r}^{s}(k)\\ E\{\phi_{i,r}^{s}(k)\}=R_{r}^{s}(k)-\mu_{i}{\tilde{I}}_{r}^{s}(k)+\lambda_{i}{\tilde{N}}_{i,r}^{s} \end{array}$$

where the model is for a single satellite. Similar expressions can be seen in [19,27] with the single-difference operator between satellites. Here, we focus on using raw measurements without any difference and combination operator in observation domain. The epoch-wise solution of the ambiguity term is simply computed as follows:

(6) $$x_{k}^{s}=H\;z_{k}^{s}$$

where the phase and code measurements can be written in a vector form as $z_{k}^{s}:={\left[\begin{array}{cccc} p_{1,r}^{s}(k) & p_{2,r}^{s}(k) & \phi_{1,r}^{s}(k) & \phi_{2,r}^{s} \end{array}(k)\right]}^{\;T}$ and the parameters that we are interested is ambiguity term $x_{k}^{s}:={\left[\begin{array}{cc} {\tilde{N}}_{1,r}^{s} & {\tilde{N}}_{2,r}^{s} \end{array}\right]}^{\;T}$. **H** is the coefficient matrix derived from Equation (5) only for ambiguity parameters as follows:

$$Η:=\left[\begin{array}{cccc} -\frac{1}{\lambda_{1}}\frac{\mu_{2}+\mu_{1}}{\mu_{2}-\mu_{1}} & \frac{1}{\lambda_{1}}\frac{2\mu_{1}}{\mu_{2}-\mu_{1}} & \frac{1}{\lambda_{1}} & 0\\ -\frac{1}{\lambda_{2}}\frac{2\mu_{2}}{\mu_{2}-\mu_{1}} & \frac{1}{\lambda_{2}}\frac{\mu_{2}+\mu_{1}}{\mu_{2}-\mu_{1}} & 0 & \frac{1}{\lambda_{2}} \end{array}\right]$$

The ionosphere and geometry terms are eliminated, which is the origin that the term of GF actually comes from.

The stochastic model for the observation is given as follows:

(7) $$Q_{z_{k}^{s}}=diag\{\sigma {\left(e^{s}\right)}_{p}^{2},\sigma {\left(e^{s}\right)}_{p}^{2},\sigma {\left(e^{s}\right)}_{\varphi }^{2},\sigma {\left(e^{s}\right)}_{\varphi }^{2}\}$$

where *e^s^* is the elevation of the satellite. According to the *error propagation law*, the variance-covariance of ambiguity terms can be derived as:

(8) $$Q_{x_{k}^{s}}^{}=HQ_{z_{k}^{s}}H^{T}$$

where Equations (6) and (8) are used to compute the epoch-wise L1 and L2 ambiguities and their variance-covariance. However, the epoch-wise observational model has no redundancy. The solution is rather noisy as it is purely dominated by the noise of code measurements. To increase the accuracy, an averaging filter is implemented using multi-epoch solutions in a recursive way. Let us notate the averaging solution as follows:

(9) $${\hat{x}}_{k}^{s}:={\left[\begin{array}{cc} {\hat{\tilde{N}}}_{1,r}^{s} & {\hat{\tilde{N}}}_{2,r}^{s} \end{array}\right]}^{\;T}$$

and the averaging filter starts at epoch 1 as ${\hat{x}}_{1}^{s}=x_{1}^{s}$ and $Q_{{\hat{x}}_{1}^{s}}=Q_{x_{1}^{s}}$. 

Assume that the filtering solution at the epoch *k*−1 is denoted as ${\hat{x}}_{k-1}^{s}$ with its covariance matrix $Q_{{\hat{x}}_{k-1}^{s}}$, the variance-covariance is first updated with the assumed process noise for epoch solution at *k*:

(10) $$Q_{{\hat{x}}_{k,k-1}^{s}}=Q_{{\hat{x}}_{k-1}^{s}}+Q_{w}$$

where, $Q_{w}$ is computed from the assumed process noise multiplying with time interval between epoch *k* and *k*−1, and then updated with the epoch-wise variance-covariance:

(11) $$Q_{{\hat{x}}_{k}^{s}}={\left(Q_{{\hat{x}}_{k,k-1}^{s}}^{-1}+Q_{x_{k}^{s}}^{-1}\right)}^{-1}$$

with the parameter being also updated with the epoch-wise estimate and its variance-covariance:

(12) $${\hat{x}}_{k}^{s}={\hat{x}}_{k-1}^{s}+Q_{{\hat{x}}_{k}^{s}}Q_{x_{k}^{s}}^{-1}(x_{k}^{s}-{\hat{x}}_{k-1}^{s})$$

where elements of ${\hat{x}}_{k}^{s}$ and $Q_{{\hat{x}}_{k}^{s}}$ are used to derive the WL ambiguity, i.e.:

(13) $${\hat{\tilde{N}}}_{wl,r}^{s}={\hat{\tilde{N}}}_{1,r}^{s}-{\hat{\tilde{N}}}_{2,r}^{s}$$

with its formal error being derived:

(14) $$\sigma_{{\hat{\tilde{N}}}_{wl,r}^{s}}=\sqrt{\sigma_{{\hat{\tilde{N}}}_{1,r}^{s}}^{2}+\sigma_{{\hat{\tilde{N}}}_{2,r}^{s}}^{2}-2\cdot \sigma_{{\hat{\tilde{N}}}_{1,r}^{s},{\hat{\tilde{N}}}_{2,r}^{s}}}$$

where $\sigma_{{\hat{\tilde{N}}}_{1,r}^{s}}^{2},\;\sigma_{{\hat{\tilde{N}}}_{2,r}^{s}}^{2},\;\sigma_{{\hat{\tilde{N}}}_{1,r}^{s},{\hat{\tilde{N}}}_{2,r}^{s}}$ are taken from $Q_{{\hat{x}}_{k}^{s}}$.

## 3. WL Ambiguities Derived from the GB Model

To derive the ambiguities from the GB model, we assume that the station coordinates $\left(X_{r},Y_{r},Z_{r}\right)$, satellite orbit $\left(X^{s}(k),Y^{s}(k),Z^{s}(k)\right)$ and clock errors $\delta \;t_{if}^{s}(k)$ are precisely known. As we know that the satellite clock errors are calculated from ionosphere-free combination observations [2]:

(15) $$\delta \;t_{if}^{s}(k)=\delta \;t^{s}(k)+\frac{\mu_{2}}{\mu_{2}-\mu_{1}}d_{p_{1}}^{s}-\frac{\mu_{1}}{\mu_{2}-\mu_{1}}d_{p_{2}}^{s}$$

and the computed range between satellite and the known station coordinates is:

$$\rho_{r}^{s}(k)=\sqrt{{\left\{X^{s}(k)-X_{r}\right\}}^{2}+{\left\{Y^{s}(k)-Y_{r}\right\}}^{2}+{\left\{Z^{s}(k)-Z_{r}\right\}}^{2}}$$

Add Equation (15) to Equation (1), insert $\rho_{r}^{s}(k)$ the left side, and define the observed minus computed values as:

(16) $$\begin{array}{l} \delta \;p_{i,r}^{s}(k):=p_{i,r}^{s}(k)-\rho_{r}^{s}(k)+\delta \;t_{if}^{s}(k)\\ \delta \;\varphi_{i,r}^{s}(k):=\varphi_{i,r}^{s}(k)-\rho_{r}^{s}(k)+\delta \;t_{if}^{s}(k) \end{array}$$

resulting in the following:

(17) $$\begin{array}{l} E\{\delta \text{​}p_{i,r}^{s}(k)\}=m_{r}^{s}T_{r}+t_{r}(k)+\mu_{i}{\tilde{I}}_{r}^{s}(k)\\ E\{\delta \;\varphi_{i,r}^{s}(k)\}=m_{r}^{s}T_{r}+t_{r}(k)-\mu_{i}{\tilde{I}}_{r}^{s}(k)+\lambda_{i}^{}{\tilde{N}}_{i,r}^{s} \end{array}$$

where the ionosphere and ambiguity terms are defined as the same as in Equations (3) and (4), respectively, and the receiver clock term is defined as:

$$t_{r}(k):=\delta \text{​}t_{r}(k)+\frac{\mu_{2}}{\mu_{2}-\mu_{1}}d_{p_{1},r}-\frac{\mu_{1}}{\mu_{2}-\mu_{1}}d_{p_{2},r}$$

The observation equations of all observed satellites in the matrix-vector form can be expressed as:

(18) $$y_{k}=A_{k}\;{\tilde{x}}_{k}+\varepsilon$$

where ${\tilde{x}}_{k}^{}:={\left[\begin{array}{ccccc} T_{r} & t_{r}(k) & I_{r}(k) & N_{1,r}^{} & N_{2,r}^{} \end{array}\right]}^{\;T}$ with $I_{r}(k):={\left[\begin{array}{cccc} {\tilde{I}}_{r}^{1}(k) & {\tilde{I}}_{r}^{2}(k) & \cdots & {\tilde{I}}_{r}^{m}(k) \end{array}\right]}^{\;T}$ and $N_{i,r}(k):={\left[\begin{array}{cccc} {\tilde{N}}_{i,r}^{1} & {\tilde{N}}_{i,r}^{2} & \cdots & {\tilde{N}}_{i,r}^{m} \end{array}\right]}^{\;T}$, $y_{k}:={\left[\begin{array}{cccc} P_{1,r} & P_{2,r} & \Phi_{1,r} & \Phi_{2,r} \end{array}\right]}^{\;T}$ with $P_{i,r}:={\left[\begin{array}{cccc} \delta \;p_{i,r}^{1} & \delta \;p_{i,r}^{2} & \cdots & \delta \;p_{i,r}^{m} \end{array}\right]}^{\;T}$ and $\Phi_{i,r}:={\left[\begin{array}{cccc} \delta \;\varphi_{i,r}^{1} & \delta \;\varphi_{i,r}^{2} & \cdots & \delta \;\varphi_{i,r}^{m} \end{array}\right]}^{\;T}$; $A_{k}\;$ is the design matrix with its elements being formed with the coefficients in Equation (17) for all visible satellites. 

The stochastic model is expressed as:

(19) $$\varepsilon \sim N(0,\;Q_{y_{k}})$$

where $Q_{y_{{}_{k}}}={\left[\begin{array}{cccc} Q_{P_{1}} & Q_{P_{2}} & Q_{\Phi_{1}} & Q_{\Phi_{2}} \end{array}\right]}^{}$, with $Q_{P_{1}}=Q_{P_{2}}=diag\left\{\begin{array}{cccc} \sigma {\left(e^{1}\right)}_{p}^{2} & \sigma {\left(e^{2}\right)}_{p}^{2} & \cdots & \sigma {\left(e^{m}\right)}_{p}^{2} \end{array}\right\}$ and $Q_{\Phi_{1}}=Q_{\Phi_{2}}=diag\left\{\begin{array}{cccc} \sigma {\left(e^{1}\right)}_{\varphi }^{2} & \sigma {\left(e^{2}\right)}_{\varphi }^{2} & \cdots & \sigma {\left(e^{m}\right)}_{\varphi }^{2} \end{array}\right\}$.

It is noticed that the observation Equation (18) has redundancy when the number of satellites is larger than three, which is easy to achieve even when only GPS satellites are employed. The state transition equation is given by:

(20) $${\tilde{x}}_{k}=B\;{\tilde{x}}_{k-1}+w_{}^{}$$

where $B:=diag{\left\{\begin{array}{ccccc} 1 & 0 & 0_{m} & e_{m}^{} & e_{m}^{} \end{array}\right\}}^{\;}\;$ with $0_{m}:=\left[\begin{array}{cccc} 0 & 0 & \cdots & 0 \end{array}\right]$,$e_{m}:={\left[\begin{array}{cccc} 1 & 1 & \cdots & 1 \end{array}\right]}^{\;}$ and $w\sim N(0,\;Q_{w})$, which links the troposphere and ambiguity parameters with that of previous epochs.

Assume that the filtering solution at the epoch k−1 is denoted as ${\hat{\tilde{x}}}_{k-1}^{}$ with its covariance matrix $Q_{{\hat{\tilde{x}}}_{k-1}}$, the solution at the epoch *k* is computed as:

(21) $${\hat{\tilde{x}}}_{k}={\hat{\tilde{x}}}_{k,k-1}+K_{k}(y_{k}-A_{k}\;{\hat{\tilde{x}}}_{k,k-1})$$

with its covariance matrix being computed as:

(22) $$Q_{{\hat{\tilde{x}}}_{k}}=(I-K_{k}A_{k})Q_{{\hat{\tilde{x}}}_{k,k-1}}$$

where ${\hat{\tilde{x}}}_{k,k-1}=B\;{\hat{\tilde{x}}}_{k-1}$, and its covariance matrix as $Q_{{\hat{\tilde{x}}}_{k,k-1}}=BQ_{{\hat{\tilde{x}}}_{k-1}}B^{T}+Q_{w}$, and the gain matrix is computed as $K_{k}=Q_{{\hat{\tilde{x}}}_{k,k-1}}A_{k}^{T}{\left(A_{k}^{}Q_{{\hat{\tilde{x}}}_{k,k-1}}A_{k}^{T}+Q_{y_{k}}\right)}^{-1}$.

The L1 and L2 ambiguities with their covariance matrix can be used to derive the GB WL ambiguity and its formal error as Equations (13) and (14) for their GF counterparts.

## 4. Impact of Orbit and Clock Residual Errors on Ambiguity Estimation

From Equations (5) and (17), there is no difference between the GF and GB ambiguities in terms of parameterization. However, their estimates are different since they go through different estimation procedures. For the GF ambiguities, the geometry-related terms, including orbit and clock, are eliminated without effects. However it results in no redundancy for the epoch-wise estimates. The solution’s accuracy is dominated by the noise of code measurements. In this case, the solution with multiple epochs is computed. For the GB ambiguities, the geometry strength is higher (as there are redundancy in each epoch), the ambiguities converge quicker than the GF ones. However, the observed minus computed values are computed from the orbit and clock, which are not always precise enough. The orbit and clock residual error at the LOS remains in the observation as follows:

(23) $$\begin{array}{l} E\{\delta \text{​}p_{i,r}^{s}(k)\}=m_{r}^{s}T_{r}+t_{r}(k)+\mu_{i}{\tilde{I}}_{r}^{s}(k)+oc_{r}^{s}\\ E\{\delta \text{​}\varphi_{i,r}^{s}(k)\}=m_{r}^{s}T_{r}+t_{r}(k)-\mu_{i}{\tilde{I}}_{r}^{s}(k)+\lambda_{i}^{}{\tilde{N}}_{i,r}^{s}+oc_{r}^{s} \end{array}$$

where $oc_{r}^{s}$ indicates the slowly time-variant or time-invariant residual part of orbit and clock error combination in the LOS direction. As they are not random noise, they could be distributed to every parameters (as well as measurement residuals), but most likely to the ionosphere and ambiguity terms of the satellite as these parameters are satellite dependent. There are two cases worthy of investigation.

### 4.1. Orbit and Clock Residual Error Being Time-Constant Bias

It is possible that the orbit and clock contain a constant bias. For example, the code measurement type used in mobile side is not consistent with the code measurements used in the reference side when computing the clock products. It is assumed that the $oc_{r}^{s}$ is a constant during the whole measurement period and there are no effects between satellites. As phase measurements are assigned much higher weight than code ones (100 times smaller in terms of standard deviation), we assume no significant orbit and clock errors are attributed to phase measurement residuals. Furthermore, the ambiguities are common terms between epochs, they likely absorb the constant bias as:

(24) $$\begin{array}{l} {\tilde{\tilde{N}}}_{1,r}^{s}:\approx {\tilde{N}}_{1,r}^{s}+\frac{1}{\lambda_{1}}\frac{\mu_{1}+3\mu_{2}}{2\mu_{2}}oc_{r}^{s}\\ {\tilde{\tilde{N}}}_{2,r}^{s}:\approx {\tilde{N}}_{2,r}^{s}+\frac{1}{\lambda_{2}}\frac{3\mu_{1}+\mu_{2}}{2\mu_{1}}oc_{r}^{s} \end{array}$$

The remaining error may be distributed to the ionosphere term and the code residuals. From the analytical expression of Equation (24), the effect on the WL ambiguity term is less than one tenth of $oc_{r}^{s}$ in terms of cycle. In other words, the effect of constant bias in orbit and clock looks largely reduced. Bearing in mind that the reality can be more complex when our assumptions do not hold for all cases. In particular, the geometry changing may have influence as well. However, the effects of a constant bias analyzed here might be a minimum (i.e., a bottom line).

### 4.2. Orbit and Clock Residual Error Being Time-Variant Error

The residual orbit and clock error is rarely a constant bias in an entire tracking period, except for a mixture use of code measurements. It is very likely a time variant error that caused by the changes of LOS and the orbit error itself (as will be shown later). In this case, it is not a trivial to make a strict analytical expression, as the estimation procedure is rather complex both in current epoch and correlation between epochs. Assume that the error is directly mapped to the ambiguity terms as follows:

(25) $$\begin{array}{l} {\tilde{\tilde{N}}}_{1,r}^{s}:\approx {\tilde{N}}_{1,r}^{s}+\frac{1}{\lambda_{1}}oc_{r}^{s}\\ {\tilde{\tilde{N}}}_{2,r}^{s}:\approx {\tilde{N}}_{2,r}^{s}+\frac{1}{\lambda_{2}}oc_{r}^{s} \end{array}$$

where the effect on WL ambiguity term will be about $1.165\cdot oc_{r}^{s}$ cycle. In other words, one meter bias could bring 1.165 cycle error into the WL ambiguity. Even when the error is about 15 cm (often the case for real-time orbit and clock), the effects will be significantly up to 0.18 cycle, which is not acceptable for phase bias calibration for PPP IAR. In reality, the effects might be around (larger or smaller than) this level due to different geometry strength. This contribution will make a real-data numerical analysis in order to further understand the effects.

## 5. Experiment and Results Analysis

**Figure 1:**
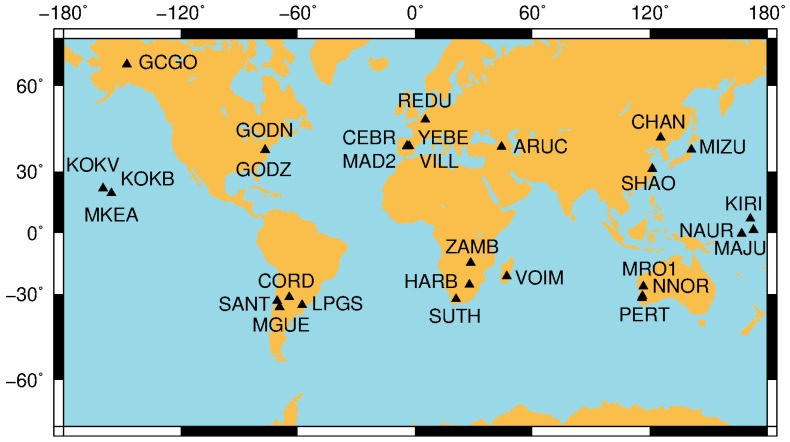
Distribution of sites that are used for this study.

In order to analyze the performance of GF and GB WL ambiguities and evaluate the orbit and clock effects on the WL ambiguities, we implemented the GF and GB models using raw observations to compute L1, L2 and WL ambiguities as well as their variance-covariance as described in Section 2 and Section 3. GNSS measurements with 1 s interval from selected IGS sites are downloaded from the IGS data base. These sites are deliberately selected and spread over main continents and the middle of the Pacific in terms of groups, see Figure 1. Some groups have sites located very nearby in order to have almost the same LOS. Two types of orbit and clock products are used in the experiment. One type is the determined orbit and clock products provided by ESA to the IGS, while the other is the real-time orbit and clock estimated as described in [15]. The interval of satellite orbit is 15 min, and the sample rate of satellite clock is 30 s for both types of products. The determined products are obtained by the post-processing mode, and their accuracy is better than that of real-time ones [15]. The standard GB PPP solutions, in parallel with the GF process, are computed using raw L1 and L2 observations. The parameters to be estimated are tropospheric zenith delay, ionosphere, receiver clock bias, and L1, L2 and then WL ambiguity terms for the GB process, while the GF process just produces the L1, L2 and then WL ambiguity terms. The positions of sites are fixed to the “ground truth”, which are computed from many days’ data with the static mode. This contribution makes use of the precise coordinates in order to determine precisely the GB-GF ambiguities by avoiding of the effects of unknown coordinates. The orbit and clock are integrated and interpolated for each epoch (1 s of data interval). Strictly speaking, the interpolation for real-time orbit and clock is not realistic in the real-time application as the future correction stream cannot be known in advance for interpolation of current epoch. However, we employ exactly the same process in order to carry out a fair comparison for both types of products by ignoring correction age effects. The elevation-dependent data weighting is used for both processes. The entire process is implemented through a strictly forward computation.

### 5.1. Analysis of GB and GF Ambiguities

**Figure 2:**
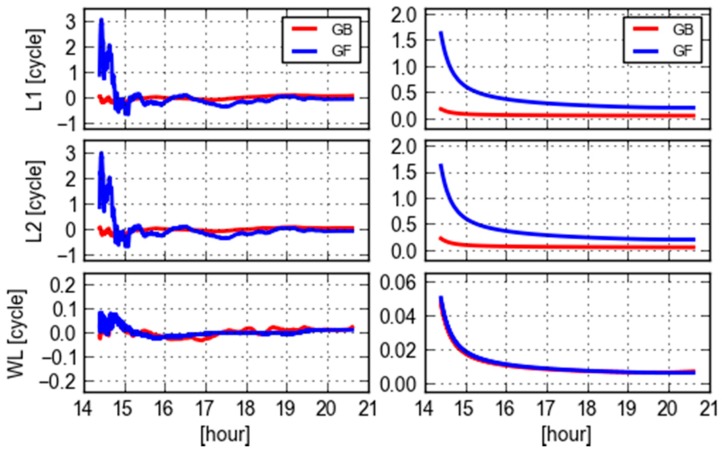
GF (blue) and GB (red) L1, L2 and WL ambiguities (**left**) and their corresponding formal errors (**right**) of PRN 09 computed from a single receiver (CEBR) observations and ESA final orbit and clock products.

This section will analyze the GB and GF ambiguities and inspect the similarity and discrepancies of GB and GF WL ambiguities. Figure 2 shows L1, L2 and WL ambiguities and their corresponding formal standard deviations for PRN 09. These ambiguities are respectively produced with the GF and GB models for site CEBR. Arbitrary offsets are removed from these ambiguities. It is obvious that both the variation of L1 and L2 GF ambiguities and their formal errors are larger than the GB counterparts, and especially they converge much slower than that of GB. However, the formal errors of WL ambiguities are very consistent and almost the same, meanwhile their estimates reveal a consistent trend despite of some small fluctuations. This confirms the analytical statement in [19] that the GF WL ambiguity precision does not differ too much from its GB counterpart. The reason is that the GF L1 and L2 ambiguities are highly correlated and even more correlated than GB ones. The WL ambiguities are therefore much precise after the differential operator, referring to (13) and (14), compared with the L1 and L2 ones. It is noticed that the GF WL ambiguities are slightly oscillated, compared with the GB ones at the first hour, but they are even stable than the GB ones afterwards. The reason is that the GB WL ambiguities may suffer some errors in orbit and clock. This will be further analyzed in other sections.

**Figure 3:**
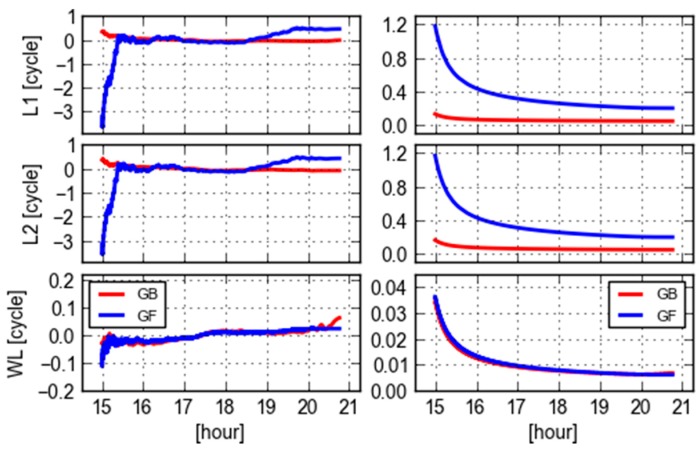
GF (blue) and GB (red) L1, L2 and WL ambiguities (**left**) and their corresponding formal errors (**right**) of PRN 09 computed using a single receiver (REDU) observations and ESA final orbit clock products.

As can be seen from Figure 3 that the results of PRN 09 computed by another single receiver REDU have similar behavior to that of CEBR, which indicates that the WL ambiguities and their formal errors have commonality for this same satellite obtained by different receivers which are located not very far away.

**Figure 4:**
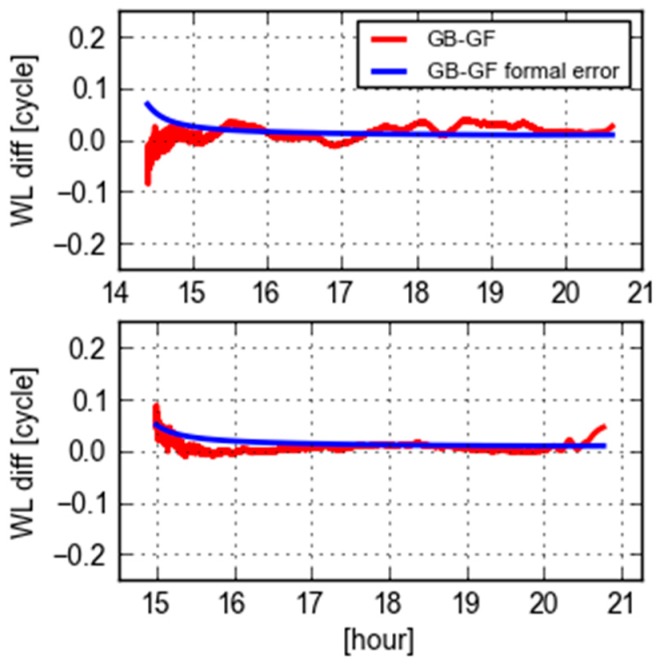
Differences of GB and GF WL ambiguities (red) and their formal errors (blue) of PRN 09 from the receivers CEBR (**top**) and REDU (**bottom**).

Figure 4 displays the differences of GB and GF WL ambiguities and their computed standard deviations derived from GB and GF ones using the error propagation law for PRN 09 at the site CEBR and REDU, respectively. The values of GB-GF are stable in between ± 0.1 cycles. The variation at the first hour is largely dominated by the GF ambiguities, while it changes due to the variation of GB ambiguities afterwards. The variations of GB-GF values are mostly within three times of the formal errors, which indicates that the formal errors statistically reflect the level of quality.

**Figure 5:**
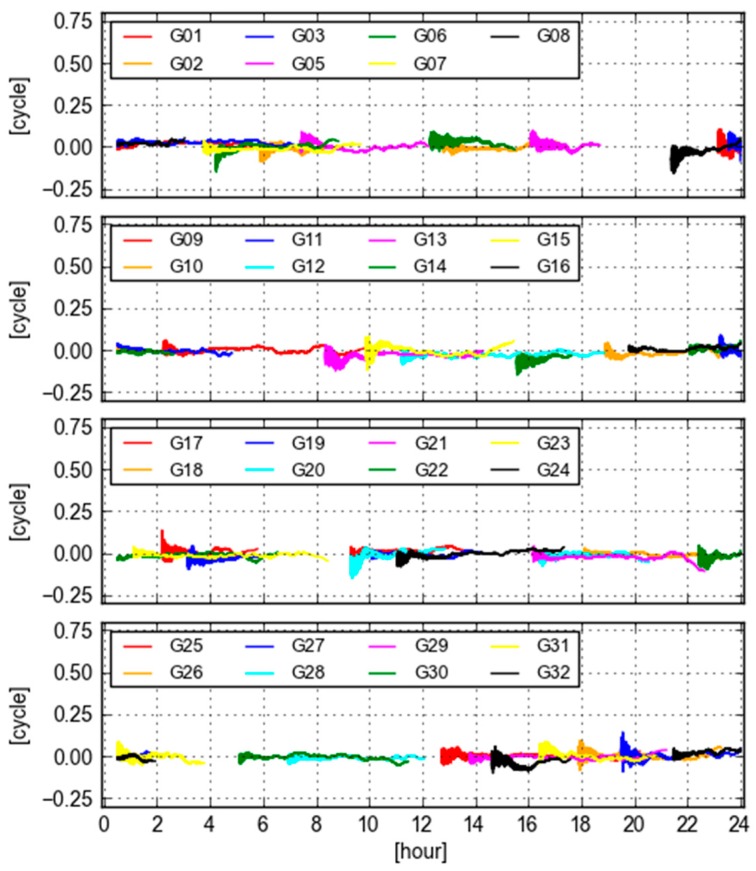
Differences of GB and GF WL ambiguities computed with ESA final orbit and clock products at site KOKV.

Figure 5 displays the values of GB-GF WL ambiguities computed with ESA final orbit and clock products at site KOKV for all visible GPS satellites. It is seen that these values are stable and close to zero. Again, the typical behavior of these time series is that they are noisy at the beginning of a tracking period, but become smooth and stable afterwards. According to the statistics, the biases are smaller than 0.05 cycle and the standard deviations of these values are smaller than 0.025 cycle. It is practically and commonly believed that the repeatability is good enough if differences of ambiguities are smaller than 0.1 cycle [7]. Therefore, we could state that the GB and GF WL ambiguities are equivalent in this case, though they are not the same.

### 5.2. Orbit and Clock Residual Error Effects

The results demonstrated in previous section are computed with the ESA final orbit and clock products. To compare with them, this section will show results that are computed with the real-time orbit and clock products. 

**Figure 6:**
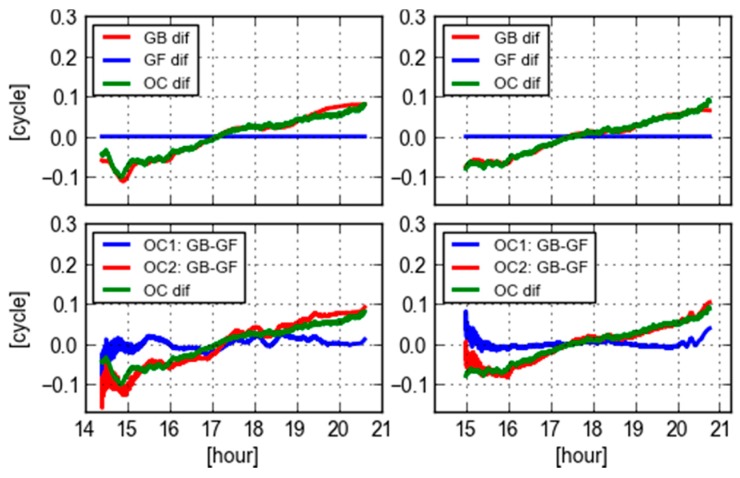
Various differences of PRN 09 WL ambiguities and orbit clock products computed at the receiver CEBR (**left**) and REDU (**right**): (1) differences of GB ambiguities computed with final and real-time orbit clock products are indicated in red in top panels; (2) differences of GF ambiguities computed with final and real-time orbit clock products are indicated in blue in top panels; (3) differences of GB and GF ambiguities computed with final orbit clock products (OC1) are indicated in blue in bottom panels; (4) differences of GB and GF ambiguities computed with real-time orbit clock products (OC2) are indicated in red in bottom panels; (5) differences of orbit clock impacts in LOS directions between final and real-time orbit clock products in green.

Figure 6 displays various differences of PRN 09 WL ambiguities and orbit clock products computed at the site CEBR and REDU, respectively. In the top panels, the GF WL differences are zeros as the orbit and clock are eliminated and have no impact on the GF ambiguity estimates. The GB WL differences are computed with final and real-time orbit and clock. In this case, everything is the same except that the orbit and clock used are different. Therefore, the GB WL differences indicate that the effects of the different orbit and clock products. To further confirm this, the differences of two types of orbit and clock are also computed for the locations of the receivers, in which the orbits are mapped into the LOS direction. The values are converted to cycles by divided with the wavelength of WL ambiguities (which is 86.2 cm). It is clear that the variations of two sets of orbit and clock differences largely coincide with the differences of GB WL ambiguities. In the bottom panels, the GB minus GF WL ambiguities used two types of orbit and clock products are shown together with the orbit and clock differences themselves. The values of GB-GF computed from the final orbit and clock (OC1: GB-GF in the figure) are more stable than that of computed from the real-time orbit and clock (OC2: GB-GF in the figure). The latter one has a clear slope in the time series, which is consistent with the slope of orbit and clock differences. In this case, it is difficult to say the GF WL ambiguities are equivalent with the GB ones. However, it is promising for the real-time users that the GB-GF WL ambiguities can be used to an indicator of the orbit and clock quality from a user’s perspective.

**Figure 7:**
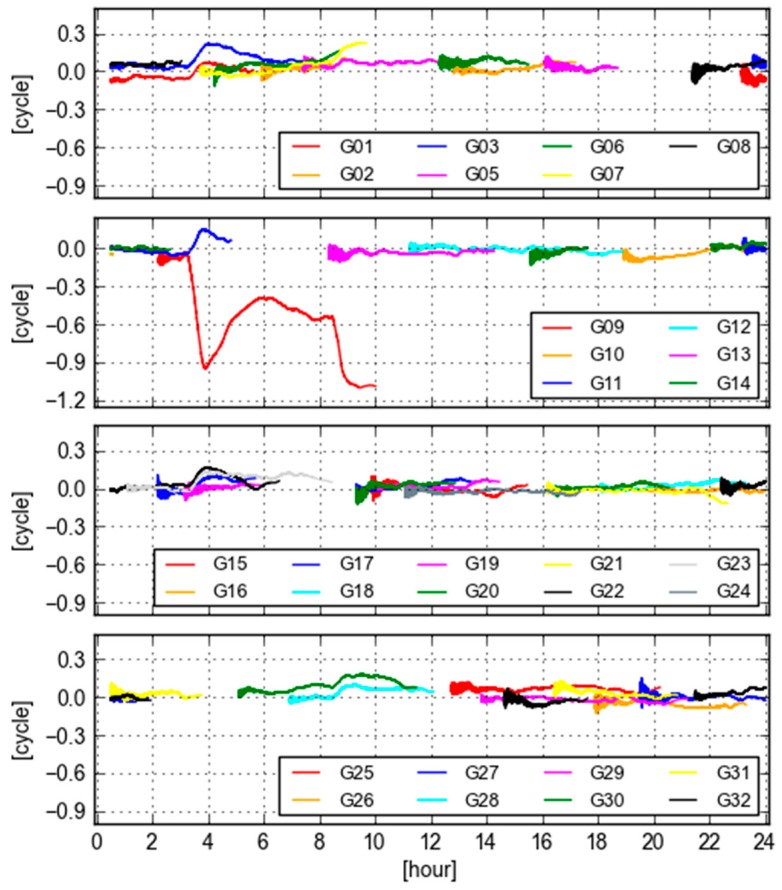
Differences of GB-GF WL ambiguities computed with real-time orbit and clock products at Site KOKV.

Figure 7 displays the values of GB-GF WL ambiguities computed with real-time orbit and clock products at site KOKV for all visible GPS satellites. During the period of 2–12 O’clock, the variations of these time series are larger than the rest of day. In particular, the largest variation is from PRN 09, which is even larger than 1.0 cycle during 9–10 O’clock. The bias and the standard deviation are −0.569 and 0.305 cycle according to the statistics. In this case, the GB and GF WL ambiguities are not equivalent.

**Figure 8:**
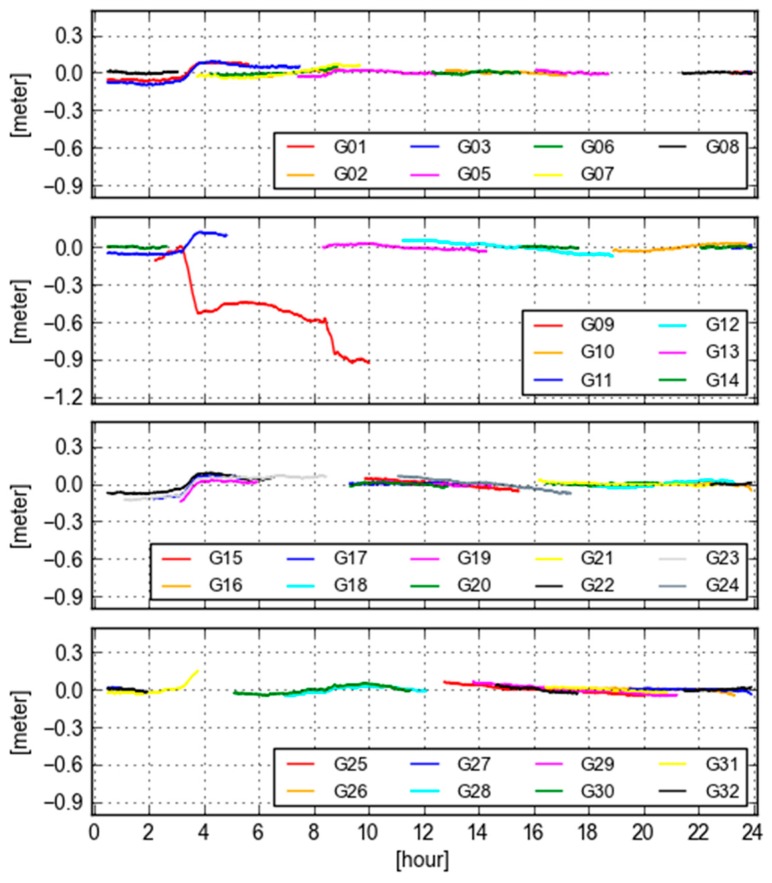
Variations of final orbit/clock and real-time orbit/clock differences projected into the LOS at Site KOKV for all visible GPS satellites.

To further investigate the cause of the variation in GB-GF WL ambiguities, we plot the variations of differences between the final and real-time orbit clock (orbit is projected into the LOS) at Site KOKV for all visible GPS satellites in Figure 8. It is clearly seen that the pattern of the orbit and clock differences is very similar with that of GB-GF ambiguities, in particular, for the satellite PRN 09. The reason for such an extreme orbit and clock behavior in real-time is likely that the orientation of the satellite (mainly IIF satellites) is estimated or predicted wrongly [22]. This issue has not been solved in real-time case. Therefore, it is valuable that the variation of GB-GF values of a receiver can be taken as a quality index of orbit clock products from users’ perspective.

**Figure 9:**
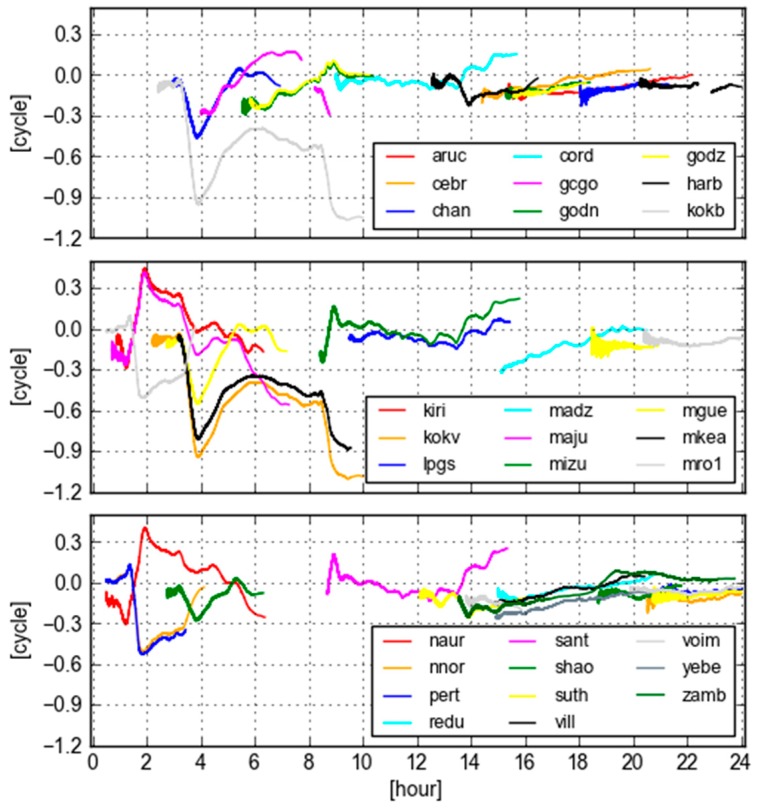
GB-GF WL ambiguities computed with real-time orbit clock products for GPS PRN 09 at all selected sites.

Figure 9 displays GB-GF WL ambiguities of PRN 09 computed with real-time orbit clock products for all selected sites. It is obvious that these time series are not stable. The figure discloses longer period of oscillations than one single site shown in Figure 7. We could also see that the variation patterns are almost the same for some pairs of sites, e.g., KOKB and KOKV, KIRI and NAUR, MIZU and SANT, MRO1 and PERT. These pairs of sites have different observations, but demonstrate the same GB-GF WL patterns, as they suffer almost the same orbit clock error. It is clear in this case that the GB and GF WL ambiguities are not equivalent.

## 6. Simulation Study of Orbit/Clock Error Effects

In order to further investigate the effects of orbit and clock errors on the WL ambiguities, we simulate two types of errors directly in the LOS for one satellite of an entire tracking period at Site KOKV: (1) a constant bias (e.g., 1.0 m); (2) time-variant errors with 0.15 m amplitude and 2 h period of sine or cosine changes. The errors are added to only PRN 30 of the ESA final orbit and clock. The GB-GF WL ambiguities are then recomputed and compared with the previous ones. The purpose for such a simulation is to see how effects of precisely “known errors” on the GB-GF ambiguities. 

**Figure 10:**
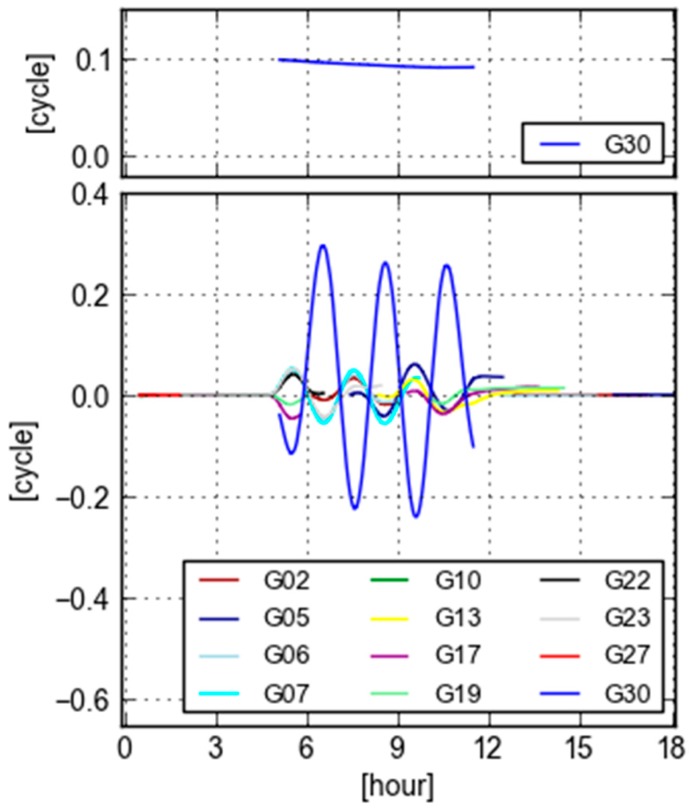
Recomputed GB-GF WL ambiguities minus the previous ones in Figure 5 with the simulated constant bias (**top**) and the time-variant errors (**bottom**) added into orbit clock of PRN 30 at Site KOKV.

Figure 10 displays differences of recomputed GB-GF WL ambiguities with respect to their counterparts without the simulated errors. The differences are purely caused by the simulated errors. The top panel displays the GB-GF WL effects of a 1.0 m constant bias is added into the PRN 30 during 5:00 h–11:30 h. The effect is about a constant bias of 0.084 cycle. There is no visible effects on other satellites. This indicates that the GB-GF WL ambiguity is not very sensible to a constant bias, which confirms the analysis in “orbit and clock residual error being constant bias”.

The bottom panel demonstrates the GB-GF WL effects after the time-variant errors are added into the PRN 30 during 5:00 h–11:30 h. Firstly, the effects of PRN 30 show also the sine or cosine behavior. Secondly, there are also similar patterns with smaller magnitude in many other satellites tracked in the same period. These effects are brought by the added errors with only 0.15 m in (absolute) size, which is much smaller than 1.0 m. This indicates that the GB-GF WL ambiguity is very sensible to time-variant errors as analyzed in Section 4.1, which provides an opportunity for real-time user to evaluate the quality of real-time orbit and clock.

## 7. Conclusions

This contribution derives L1, L2 and WL ambiguities from the GF and GB PPP model. Two types of orbit and clock products, represented different level of quality, are used to compute the WL ambiguities. The differences of GB and GF WL ambiguities are particularly analyzed. The main results and conclusions are summarized in the following statements:

The GF ambiguities are obtained on an individual satellite basis, and there is no redundancy for the epoch-wise estimation. To improve GF estimates, multiple data epochs are needed. The GB ambiguity estimation makes use of all received satellites, therefore the geometry and the redundancy, besides using multiple epochs of data, play roles on the estimation. From our implementation and experiment, there are significant differences between GB L1/L2 ambiguities and the GF ones in terms of both estimates and their precisions, however, the GB WL ambiguities do not differ much from the GF ones in terms of precision at least.

Orbit and clock have no impact on GF WL ambiguities as they are eliminated in the process, while they have effects on GB ones. Whether the GB and GF ambiguities are equivalent depends much on the quality of the used orbit and clock. In general, if GB-GF WL values are stably in between ± 0.1 cycles or within three times of their formal errors, we could state that the GB and GF WL ambiguities are equivalent. This is basically not an issue for the final orbit and clock product used in our experiment, as this type of product is obtained from post-processing.

The GB and GF WL ambiguities are not always equivalent if they are computed from the real-time orbit and clock, as the type of product is sometimes not perfect. It is found in this contribution that the variation of GB-GF WL ambiguities coincide with the orbit and clock differences between the final and real-time products. Therefore, it is promising for the real-time users that the GB-GF WL ambiguities can be directly used to indicate the orbit and clock quality from a user’s perspective.

Two types of orbit and clock errors are simulated and the effects on GB-GF WL ambiguities are analyzed. The constant bias in orbit and clock seems not have significant effects on GB-GF WL values if we look into the pattern of their time-series, though there is still a smaller size of bias presenting. This indicates that the GB-GF WL ambiguity is not very sensible to a constant bias in orbit and clock. However, the GB-GF WL ambiguity varies remarkably if the time-variant errors exist in the orbit and clock. Even the errors are not large, it is sensitively mapped into GB-GF WL ambiguities. As this type of errors is more often showing up than the constant bias in the real-time orbit and clock, the GB-GF WL ambiguity provides an opportunity for real-time users to evaluate the quality of real-time orbit and clock.
